# 遗传性蛋白C缺陷症致新生儿暴发性紫癜2例报告及文献复习

**DOI:** 10.3760/cma.j.issn.0253-2727.2022.12.012

**Published:** 2022-12

**Authors:** 袁园 段, 优 吴, 愿愿 许, 文佳 童, 丹群 金

**Affiliations:** 安徽省儿童医院重症医学科，合肥 230051 Department of Pediatric Intensive Care Unit, Anhui Children's Hospital, Hefei 230051, China

蛋白C是一种维生素K依赖性丝氨酸蛋白酶，是抗血栓形成调节系统的核心组成部分，具有抗凝和纤溶特性。蛋白C在体内以酶原形式存在，在内皮细胞表面凝血酶-血栓调节蛋白复合物的作用下激活成活化蛋C（APC）[1]，在蛋白S辅助下，通过灭活活化的凝血因子V和凝血因子Ⅷ来抑制凝血酶的产生，发挥抗凝作用，主要作用部位在微循环[2]。遗传性蛋白C缺陷症以反复弥散性血管内凝血和出血性皮肤坏死为主要表现，可伴有多器官出血或血栓形成。1981年Griffin等[2]首次报告，编码蛋白C的PROC基因发生突变可导致蛋白C含量和（或）功能异常，导致血栓性出血性疾病。现报告我院收治的2例遗传性蛋白C缺陷症致新生儿暴发性紫癜患者，旨在提高对该病的临床及基因诊断的认识。

## 病例资料

一、临床资料

例1，男，5日龄，因“发现腹部及足底瘀斑2 d”入院，系孕37周剖宫产娩出，羊水清，无窒息抢救史，胎盘未见异常，出生体重2850 g。患儿生后第3天家长发现左侧足底和右侧下腹部不规则瘀斑，生后第5天瘀斑加重，且睾丸部位出现瘀斑，入院前急诊B超提示左侧睾丸实质内未见明确彩色血流信号（不排除左侧睾丸部分坏死可能），收治泌尿外科拟手术治疗，外科医师查视患儿排除睾丸扭转，为进一步查明病因转入我科。家族史：父母及4岁姐姐体健，异卵双胞胎妹妹无类似表现；否认家族成员有中紫癜及深静脉血栓病史。入科时查体：生命体征平稳，右侧下腹部可见大小约3 cm×6 cm不规则瘀斑，周围伴红肿，部分皮肤破溃，伴少许渗液，部分皮肤表面黑色痂覆盖，左足底有一2 cm×1 cm瘀斑，双侧阴囊肿胀，阴茎及左侧阴囊皮肤颜色变黑，肢端皮温正常，双足背动脉可触及搏动。辅助检查：血常规示WBC 18.3×10^9^/L、HGB 150 g/L、PLT 84×10^9^/L、中性粒细胞66％；C反应蛋白（CRP）6.6 mg/L（参考值0～8.0 mg/L），降钙素原300 ng/L（参考值<200 ng/L）；凝血功能提示凝血酶原时间（PT）14.4 s（参考值10～14 s），活化部分凝血活酶时间（APTT）86.3 s（参考值23～40 s），纤维蛋白原（FIB）1.440 g/L（参考值1.80～4.00 g/L），凝血酶时间（TT）15.9 s（参考值14～21 s），D-二聚体33.34 mg/L（参考值0～0.5 mg/L）；血气、电解质、肝功能、肾功能、心肌酶谱、血糖正常，疱液分泌物涂片+培养、双份血培养无细菌生长。会阴部B超提示左侧睾丸实质回声明显降低，考虑左侧睾丸坏死，睾丸部分实质回声不均，双侧睾丸鞘膜积液，双侧阴囊壁肿胀。肝胆胰脾及肾上腺B超均未见异常。眼部B超提示右侧玻璃体占位，右视网膜部分脱离，右眼视乳头缺损可能；眼眶磁共振检查示右侧眼玻璃体内异常信号（考虑视网膜病伴出血可能），右视网膜脱离。皮肤软组织B超提示左侧枕部及右膝部软组织明显增厚；头颅CT脑实质无异常，双侧顶部、左侧颞顶枕部皮肤软组织肿胀。

例2，男，7日龄，因“发现后背部及双侧大腿皮肤红肿坏死2 d”入院。患儿为弃婴，出生史及家族史不详。收住新生儿外科，予万古霉素联合美罗培南抗感染，患儿皮肤瘀斑及硬肿范围进一步扩大，转入我科。入科时查体：生命体征平稳，体温37.8 °C，左额部及顶枕部皮肤可见大片瘀斑，心肺腹未见异常，双侧腹股沟、骶髂部、双侧大腿及左下肢皮肤可见大片状瘀斑，其上可见血疱，阴阜、阴囊及臀部硬肿，触痛明显，肢端皮温正常，双足背动脉可触及搏动。辅助检查：血常规示WBC 29.49×10^9^/L、HGB 124 g/L、PLT 73×10^9^/L、中性粒细胞74.2％；CRP 7.9 mg/L，降钙素原760 ng/L；凝血功能：PT 14.9 s，APTT 57.2 s，FIB 1.16 g/L，TT 13.9 s，D-二聚体80 mg/L；血气、电解质、肝功能、肾功能、心肌酶谱、血糖正常。疱液分泌物涂片+培养、血培养无细菌生长。头颅CT提示脑室系统稍扩张，周围白质区范围增广，透明隔间腔稍宽；两顶部头皮血肿；腹部增强CT提示胸腹部弥漫肿胀；双侧肾盂饱满；双侧睾丸密度不均，阴囊内低密度影。磁共振：双侧大脑半球白质区广泛异常信号，考虑广泛软化伴少许出血可能。幕上脑室扩张，四脑室饱满，透明隔间腔存在，小脑脑沟增深；磁共振血管成像（MRA）提示Wilis环显示中断、双侧大脑前中动脉远端显示不清，大脑后动脉全段显示不清；磁共振静脉成像（MRV）深静脉系统显示不清。

例1和例2患儿入院后给予积极抗感染、输注冷沉淀及凝血酶原复合物补充凝血因子，免疫球蛋白加强支持治疗，同时给予每12 h 1次输注新鲜冰冻血浆（10 ml/kg）和低分子肝素钠（100 IU/kg）皮下注射抗凝，加强皮肤护理等对症支持治疗。两例患儿经治疗后血小板均上升至正常范围，但皮肤瘀斑较前加重，复查凝血功能D-二聚体有所下降，停止新鲜冰冻血浆输注后迅速升高。例1入院第8天测蛋白C活性为0％，抗原为13.1％，蛋白S活性为119.3％，第12天家长放弃治疗出院，3 d后死亡。例2入院后第4天测蛋白C活性为2％，蛋白C抗原为15.6％，蛋白S活性为70.8％，给予持续补充新鲜冰冻血浆及抗凝治疗，患儿终因弥散性血管内凝血而死亡。

二、基因检测结果

例1检出PROC基因c.1032（exon9）C>G和c.400+5G>A 2个突变位点，验证父母均为杂合子突变，姐姐正常，异卵双胞胎妹妹为单杂合子突变（图1、2）。例2检出PROC基因c.1310（exon9）A>C、c.181（exon3）delG 2个突变位点（图3、4），未进行家系验证（患儿为弃婴）。以上4个PROC基因突变位点除c.400+5G>A外，均为首次报道。

**图1:**
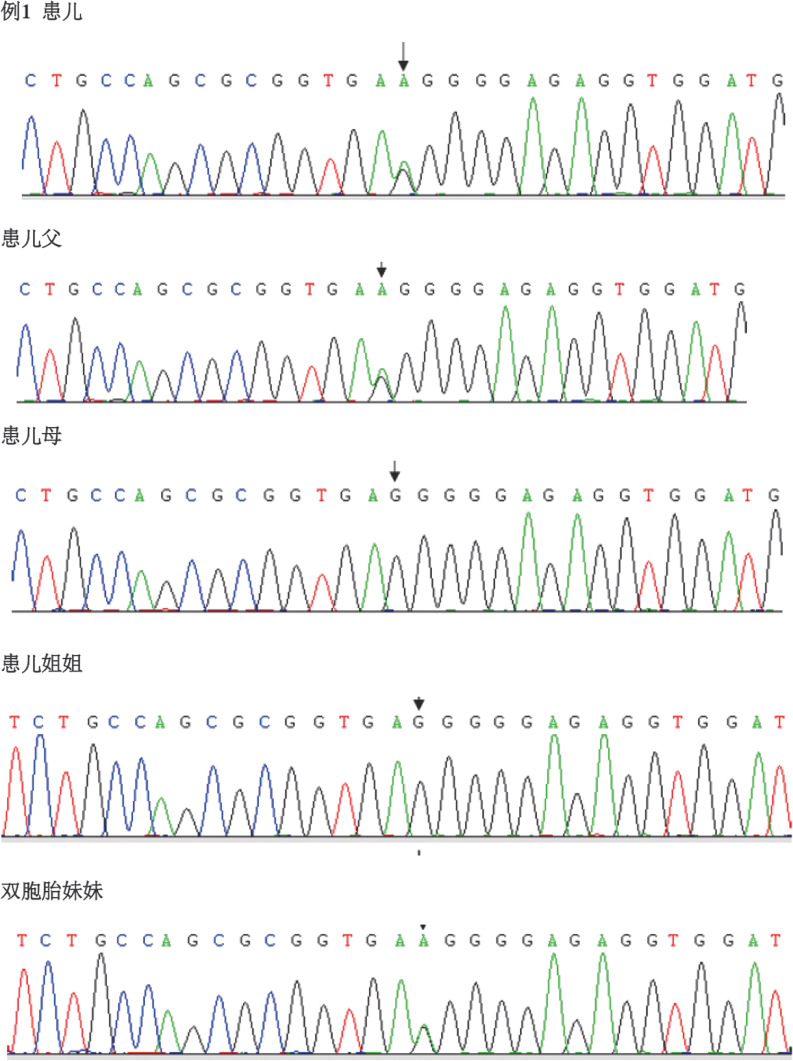
例1及其父亲、妹妹PROC基因剪切位点附近c.400+5G>A突变

**图2:**
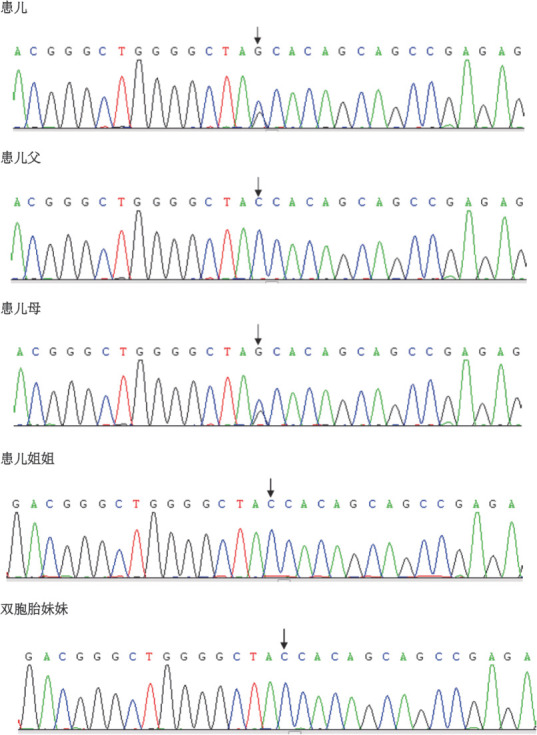
例1及其母亲基因检测结果显示PROC基因发生框移突变c.1032（exon9）C>G

**图3:**
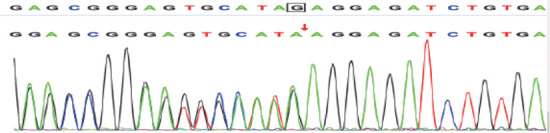
例2基因检测结果显示PROC基因发生c.181（exon3）delG突变

**图4:**
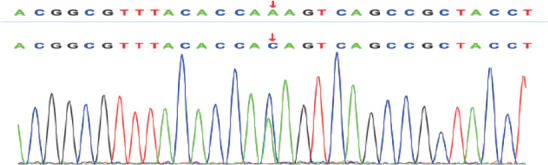
例2基因检测结果显示PROC基因9号外显子c.1310 A>C突变

## 讨论及文献复习

PROC基因定位于2q13-14，由9个外显子和8个内含子组成，根据遗传方式的不同分为杂合和纯合变异，杂合型蛋白C缺陷症多见，好发于30～40岁成人。根据蛋白C活性和抗原的检测结果分为Ⅰ型和Ⅱ型。前者由于蛋白C合成减少或功能正常的分子稳定性降低导致蛋白C活性和抗原平行降低；后者由于异常蛋白C分子合成导致蛋白C活性降低而抗原正常[3]–[4]。本组2例患儿蛋白C活性和抗原平行下降，为Ⅰ型遗传性蛋白C缺陷。

人群中无症状杂合蛋白C缺陷症的发病率为1/200～1/500，有症状杂合蛋白C缺陷症的发病率为1/16万～1/32万[5]，多在成年发病，临床上可引起反复深静脉血栓，患者血浆水平呈轻度下降，为正常人的30％～60％[6]。只有极少数患者是由纯合或更少见的复合杂合突变引起的严重蛋白C缺陷，血浆中蛋白C含量极度降低，大部分患者在婴儿时期即发生暴发性紫癜，发病率约为1/50万～1/75万[7]，患者出生后不久就会在微循环系统形成广泛血栓，多因严重脑损伤、多器官功能衰竭而死亡[5]。有研究显示PROC基因变异与暴发性紫癜相关，日本一项27例有蛋白C缺陷症的儿童回顾性研究显示，有16例表现为暴发性紫癜，9例患者存在PROC基因变异，提示PROC基因变异与暴发性紫癜密切相关[8]。本组两例患儿均在新生儿期发病，蛋白C活性极低，发生暴发性紫癜及广泛微循环血栓形成，患儿转归情况亦符合既往报道，经加深WES检测发现了4个PROC基因变异位点，其中3个为新发基因变异位点，目前尚未见报道。例1为PROC基因复合杂合突变c.1032（exon9）C>G和c.400+5G>A导致蛋白C缺陷，但其胞妹携带单点变异且无相关临床表现，符合常染色体隐性遗传方式。例2未进行家系基因分析，不能确定其遗传方式。

蛋白C缺陷症所致的广泛微血栓形成目前尚无有效根治方法，在国际上，主张联合应用蛋白C替代治疗及抗凝药物治疗[9]。蛋白C替代物主要包括新鲜冰冻血浆和蛋白C浓缩物，在蛋白C严重缺乏的患儿中，所需血浆用量大，且蛋白C半衰期短，停止血浆输注后症状仍会持续加重，在新生儿患者中大量新鲜冰冻血浆的输注可能导致液体过负荷、肺水肿及蛋白尿形成，且反复多次地输注不同供者的血浆不但增加传播病毒性疾病的风险，同时也增加了输注相关的过敏反应的概率[10]。

蛋白C浓缩物是从人体血浆中提取的并经高度纯化得到，进行病毒灭活，具有良好的有效性及安全性。一项多中心研究[11]显示蛋白C浓缩物治疗暴发性紫癜和急性血栓形成有效率高达95％，且无明显不良反应，经治疗后所有患者的D-二聚体迅速下降。临床研究证实蛋白C浓缩物对严重先天性蛋白C缺乏症的治疗效果优于新鲜冰冻血浆和其他抗凝血药物[12]。而在日本的27例患儿中，18例接受蛋白C浓缩物的治疗，其中2例因感染而死亡，而幸存者中8例遗留神经系统后遗症[8]，蛋白C浓缩物目前在中国尚未上市。

儿童蛋白C缺陷中常用的抗凝药物为低分子肝素及华法林。低分子肝素需静脉用药，且易发生肝素诱导的血小板减少症。华法林治疗过程中需监测凝血功能，使其在儿童中的应用受限。有研究指出直接口服抗凝药物可能成为治疗儿童蛋白C缺陷引起深静脉血栓形成的新手段[13]。我们所报道的2例患儿应用新鲜冰冻血浆联合低分子肝素钠抗凝，却没有起到预期效果，仍有反复血栓形成，分析原因可能与血浆蛋白C含量严重缺乏相关。

肝移植是一种成熟的治疗先天性严重蛋白C缺陷的方法，与终身输注蛋白C浓缩物相比，具有明显的经济优势，且避免了复发性凝血功能紊乱及器官脏器出血和血栓形成的风险。在国外，儿童肝移植大多数具有良好的长期生存率[14]–[15]。国内目前没有本病进行肝移植的相关报道。

PROC基因缺陷患者是否出现临床症状是由环境因素（妊娠、外伤、服用避孕药、长期制动等）与基因突变共同作用的结果[16]。对于蛋白C缺陷患者来说，避免使用避孕药、防止各种创伤的发生是预防深静脉血栓形成的重要措施，对于长期卧床的患者应鼓励床边康复、适度进行肌肉训练。
